# Elucidating the structure of an infectious protein

**DOI:** 10.1371/journal.ppat.1006229

**Published:** 2017-04-13

**Authors:** Markus Zweckstetter, Jesús R. Requena, Holger Wille

**Affiliations:** 1 Deutsches Zentrum für Neurodegenerative Erkrankungen, Göttingen, Germany; 2 Max Planck Institute for Biophysical Chemistry, Göttingen, Germany; 3 University Medical Center Göttingen, University of Göttingen, Göttingen, Germany; 4 CIMUS Biomedical Research Institute, University of Santiago de Compostela-IDIS, Santiago de Compostela, Spain; 5 Department of Biochemistry, University of Alberta, Edmonton, Alberta, Canada; 6 Centre for Prions and Protein Folding Diseases, University of Alberta, Edmonton, Alberta, Canada; Washington University School of Medicine, UNITED STATES

## What are infectious proteins?

The infectious isoform of the mammalian prion protein, PrP^Sc^, was the first protein to be identified as an infectious protein [1] (Table 1). PrP^Sc^ can be transmitted both from cell-to-cell and between animals or individuals and causes an invariably fatal, neurodegenerative disease [2]. Fungal prions, which are unrelated to the mammalian prion protein, convey cytosolic inheritance based on different protein folding states and are transmitted from mother to daughter cell during cell division or in the course of cytoplasmic fusion events [3,4]. In recent years, other neurodegenerative diseases, such as Alzheimer disease and Parkinson disease, were also recognized as being spread by cell-to-cell transmission of protein aggregates, although the infectivity of these particles is a matter of debate [5]. In these cases, proteins other than the prion protein (e.g., Aβ, microtubule-associated protein tau, α-synuclein, etc.) are prone to misfolding and aggregation (Table 1) and were subsequently labeled as prions, prionoids, or prion-like proteins, depending on the preferences of the authors and reflecting the open question with respect to infectivity [6].

**Table 1:** Infectious prions and prion-like proteins.

Diseases	Protein name		Amyloid deposits	Transmissibility	
	Healthy state/precursor protein	Infectious state		Cell-to-cell	Between individuals
Prion diseases (e.g., Kuru, Creutzfeldt-Jakob disease, bovine spongiform encephalopathy, chronic wasting disease, scrapie, etc.)	PrP^C^	PrP^Sc^	yes	yes	yes
Yeast prion^1^	Ure2p	[URE3]	yes	yes^2^	
Yeast prion^1^	Sup35p	[PSI+]	yes	yes^2^	
Yeast prion^1^	Rnq1p	[PIN+]	yes	yes^2^	
Heterokaryon incompatibility^1^	HET-s	[HET-s]	yes	yes	yes
Alzheimer disease	APP	Aβ	yes	yes	no^4^
Alzheimer disease & tauopathies	Tau	PHF-Tau	yes	yes	no^4^
Parkinson disease	α-synuclein^3^	α-synuclein^3^	yes	yes	no^4^
Lou Gehrig disease	SOD1^3^	SOD1^3^	yes	yes	no
Transthyretin amyloidses (many different forms)	transthyretin	transthyretin amyloid	yes	extra-cellular	no^4^
Huntington disease	huntingtin	huntingtin	no	yes	no

^1^ not a disease, but a metabolic/mating-type phenotype

^2^ unicellular organism, transmission occurs during cell division from mother to daughter cell

^3^ other proteins have also been implicated in these diseases

^4^ evidence suggests the possibility for transmission through iatrogenic or environmental mechanisms, but these claims are still under vigorous discussion

In most cases, these proteins form amyloid or amyloid-like fibrils, but other aggregation states, such as oligomers, amorphous aggregates, and 2-D crystals, have been observed. The insolubility and aggregated nature of amyloids and infectious prion particles complicates their structural characterization by X-ray crystallography, etc., but circular dichroism (CD) and Fourier-transform infrared (FTIR) spectroscopies indicated that infectious proteins generally contain predominantly β-sheets [2,5]. Therefore, often, a variety of techniques are used in combination to elucidate the structure of these infectious proteins. Those that have provided the most useful high-resolution results will be discussed in this article.

## Five approaches

### Electron microscopy and related techniques

Negative stain electron microscopy is widely used to characterize the aggregation state of amyloids and other polymers. It is a fast and straightforward tool to assess aggregate morphology and to measure their size in two dimensions but is limited to low-resolution observations. Height measurements are difficult to make via this approach and are more reliably done through scanning probe microscopies (also known as atomic force microscopy [AFM]). Protein aggregates that display intrinsic symmetry, such as helical amyloid fibrils or 2-D crystals, can be used to extract more detailed information about the aggregated protein (e.g., [7,8]). In these cases, image processing takes advantage of the repeating structure and can extract molecular details through averaging that are not readily visible. Electron tomography can rapidly provide 3-D tomograms of the observed specimens, but the dose fractionation that is necessary to collect the different view angles limits the resolution of the reconstructed volumes [9,10].

**Fig 1:**
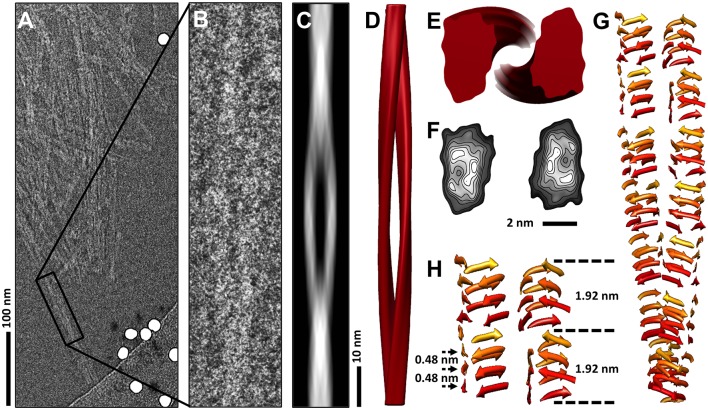
Electron cryomicroscopy analysis of infectious prion protein amyloid fibrils. **(A)** Section of a cryo electron micrograph showing prion fibrils lacking the glycosylphosphatidylinositol (GPI) anchor. A single isolated and twisted fibril used for the 3-D reconstruction is enclosed by a black box. **(B)** Close-up view of the isolated prion fibril. **(C)** Reprojected image of the 3-D fibril map for comparison with the unprocessed image (B). **(D)** 3-D reconstruction of the GPI-anchorless prion fibril. **(E)** Cross section of the reconstructed fibril showing two distinct protofilaments. **(F)** Contoured density maps of the cross section with lines contoured at increasing levels of 0.125 σ. **(G)** Cartoon depicting the proposed configuration of the polypeptide chains in the prion fibril. Please note that this is not an atomistic model. **(H)** Close-up view of the possible ß-sheet stacking in a four-rung ß-solenoid architecture for illustration purposes only. Different colors represent different ß-solenoid rungs. Characteristic distances of the four-rung ß-solenoid architecture are labeled. Figure adapted from [10].

High-resolution electron microscopy studies require the use of cryo low-dose imaging techniques. With the advent of direct electron detectors, unprecedented structural detail can be visualized, which, under optimal conditions, can reach atomic resolution [11,12]. The added sensitivity that is provided by these new detectors is revolutionizing electron cryomicroscopy and the structural details that can be obtained from even challenging samples, such as protein aggregates (Fig 1) [10]. However, the structural heterogeneity that is commonly seen with protein aggregates often limits the resolution that can be obtained, and this also applies to individual amyloid fibrils of infectious prions [10,13].

### Diffraction techniques

Protein aggregates, including those composed of infectious proteins, are also amenable to structural analyses via diffraction techniques, such as small angle X-ray scattering (SAXS), X-ray crystallography, and X-ray fiber diffraction. The latter technique is often used to detect the characteristic 4.8 Å cross-β signature of amyloid fibrils [14], which is a defining criterion commonly used in biophysics for the term “amyloid.” The need to achieve sufficient sample orientation is an ongoing challenge for X-ray fiber diffraction analyses of amyloid fibrils, but well-oriented samples can reveal the necessary structural details to define the molecular dimensions and structural architecture of different amyloid forms [15,16]. X-ray crystallography has more stringent demands, as it requires the protein to form well-ordered 3-D crystals, which is nearly impossible to achieve except with small amyloidogenic peptides [17].

In contrast, SAXS provides a measure of the sample/aggregate size without the need for sample orientation. In fact, the random orientation of the protein aggregates in solution allows calculation of the overall aspect ratio of the aggregate. Therefore, a sufficiently dispersed sample can provide molecular or protein aggregate dimensions via the radius of gyration [18].

### Nuclear magnetic resonance spectroscopy

A large array of spectroscopic techniques has been applied to the study of protein aggregates, including infectious prions. One particularly powerful technique is nuclear magnetic resonance (NMR), because it provides single-residue resolution and can be applied to both soluble species and insoluble aggregates. In case of insoluble particles, the slow tumbling time of the aggregates has to be taken into account. Therefore, solution-state NMR is best combined with hydrogen/deuterium exchange assays, in which the exchange rate of hydrogen atoms, which participate in peptide bonds, with the solvent strongly depends on the local secondary structure in the aggregate. In particular, hydrogen atoms within β-strands participate in relatively stable hydrogen bonds and exchange very slowly. This makes hydrogen/deuterium exchange coupled with solution NMR spectroscopy a powerful tool to identify the location of the regular secondary structure elements in prion aggregates [19].

The most powerful experimental technique to characterize heterogeneous protein aggregates and infectious prions to date is solid-state NMR spectroscopy [20,21]. Solid-state NMR spectroscopy has now reached a level at which it can reliably determine the 3-D structure of single molecules in amyloid fibrils [22–25]. In combination with information from other techniques, such as electron microscopy and modeling, this approach can also provide insights into the higher order arrangements of molecules in prion aggregates [26,27].

### Mass spectrometry

Mass spectrometry analysis of peptide fragments obtained under denaturing, exchange-quenching conditions has been used to assess the global exchange of short stretches of a given protein. Application of such an approach to GPI-anchorless PrP^Sc^ showed an overall very low rate of exchange of a stretch spanning from position ~81 to ~226, which is suggestive of a high content of β-sheet secondary structure and tight packing. Slightly higher exchange of some short regions within this stretch suggests the presence of short loops connecting short β-strands [28]. Similar results have been obtained more recently for infectious, recombinant PrP^Sc^, suggesting a common structure for all infectious PrP^Sc^ forms [29], which is very different from that of noninfectious recombinant amyloids [28].

### Chemical probes

Chemical probes have been successfully used to obtain structural information of proteins difficult to study by other means. Typical approaches include surface labeling, which provides information about accessibility of specific amino acids, and cross-linking with bifunctional reagents, which provides upper limits on the distance between pairs of accessible residues. Identification of modified sites is typically achieved by mass spectrometry after tryptic digestion [30].

Surface labeling of PrP^Sc^ with tyrosine-specific reagents showed that its C-terminal region has suffered a very substantial structural rearrangement, contrary to the hypothesis of conserved C-terminal α-helices [31]. PrP^Sc^ has also been probed with cross-linking reagents. Experiments using bis (sulfosuccinimidyl) suberate (BS^3^) showed that the amino termini of successive PrP 27–30 units in a PrP 27–30 stack are within 11.4 Å [32]. While such a distance constraint was interpreted at the time as a limitation to the maximum number of rungs in the PrP^Sc^ β-solenoid, it is fully compatible with head-to-head stacking of PrP^Sc^ units [10].

In summary, chemical probing should be seen as a complement to other techniques that provide a general view of the architecture of infectious proteins. Recent advances in sensitivity and accuracy of mass spectrometry methods, such as Fourier-transform instruments and chemical footprinting with synchrotron radiation, has opened up exciting new possibilities [30].

## Conclusion

Recent technological advances have provided a wealth of data on the structures of pathologically aggregated, infectious proteins involved in Alzheimer disease, Parkinson disease, and the prion diseases. In the former cases, the structure of the aggregated proteins (Aβ and α-synuclein) were found to adopt an in-register β-sheet structure [24,25]. In contrast, for the archetypical prion diseases (PrP^Sc^), a four-rung β-solenoid architecture was observed [10,15], in agreement with lower-resolution approaches [18,28,31,32]. The ability to generate disease-relevant protein conformers in vitro, in combination with solid-state NMR and other analysis techniques, was crucial for determining the high-resolution structures of misfolded Aβ and α-synuclein [24,25]. A similar approach may provide high-resolution structural information about PrP^Sc^ in the future.

## References

[1] Prusiner SB (1982) Novel proteinaceous infectious particles cause scrapie. Science 216: 136–144. 6801762 10.1126/science.6801762

[2] Colby DW, Prusiner SB (2011) Prions. Cold Spring Harb Perspect Biol 3: a006833 10.1101/cshperspect.a006833 21421910 PMC3003464

[3] Wickner RB (1994) [URE3] as an altered URE2 protein: evidence for a prion analog in Saccharomyces cerevisiae. Science 264:566–569. 7909170 10.1126/science.7909170

[4] Coustou V, Deleu C, Saupe S, Begueret J (1997) The protein product of the het-s heterokaryon incompatibility gene of the fungus Podospora anserina behaves as a prion analog. Proc Natl Acad Sci U S A 94: 9773–9778. 9275200 10.1073/pnas.94.18.9773 PMC23266

[5] Jucker M, Walker LC (2015) Neurodegenerative diseases: Expanding the prion concept. Annu Rev Neurosci 38: 87–103. 10.1146/annurev-neuro-071714-033828 25840008 PMC4803040

[6] Prusiner SB (2013) Biology and genetics of prions causing neurodegeneration. Annu Rev Genet 47: 601–623. 10.1146/annurev-genet-110711-155524 24274755 PMC4010318

[7] Serpell L (2014) Amyloid structure. Essays Biochem 56: 1–10. 10.1042/bse0560001 25131583

[8] Stahlberg H, Biyani N, Engel A (2015) 3D reconstruction of two-dimensional crystals. Arch Biochem Biophys 581: 68–77. 10.1016/j.abb.2015.06.006 26093179

[9] Terry C, Wenborn A, Gros N, Sells J, Joiner S, et al (2016) Ex vivo mammalian prions are formed of paired double helical prion protein fibrils. Open Biol 6, 160035 10.1098/rsob.160035 27249641 PMC4892434

[10] Vázquez-Fernández E, Vos MR, Afanasyev P, Cebey L, Sevillano AM, et al (2016) The structural architecture of an infectious mammalian prion using electron cryomicroscopy. PLoS Pathog 12, e1005835 10.1371/journal.ppat.1005835 27606840 PMC5015997

[11] Carroni M, Saibil HR (2016) Cryo electron microscopy to determine the structure of macromolecular complexes. Methods 95: 78–85. 10.1016/j.ymeth.2015.11.023 26638773 PMC5405050

[12] Cheng Y (2015) Single-particle cryo-EM at crystallographic resolution. Cell 161: 450–457. 10.1016/j.cell.2015.03.049 25910205 PMC4409662

[13] Mizuno N, Baxa U, and Steven AC (2011) Structural dependence of HET-s amyloid fibril infectivity assessed by cryoelectron microscopy. Proc Natl Acad Sci U S A 108: 3252–3257. 10.1073/pnas.1011342108 21300906 PMC3044374

[14] Eisenberg D and Jucker M (2012) The amyloid state of proteins in human diseases. Cell 148: 1188–1203. 10.1016/j.cell.2012.02.022 22424229 PMC3353745

[15] Wille H, Bian W, McDonald M, Kendall A, Colby DW, et al (2009) Natural and synthetic prion structure form X-ray fiber diffraction. Proc Natl Acad Sci U S A 106: 16990–16995. 10.1073/pnas.0909006106 19805070 PMC2761340

[16] Wan W, Wille H, Stöhr J, Kendall A, Bian W, et al (2015) Structural studies of truncated forms of the prion protein PrP. Biophys J 108: 1548–1554. 10.1016/j.bpj.2015.01.008 25809267 PMC4375555

[17] Sawaya MR, Sambashivan S, Nelson R, Ivanova MI, Sievers SA, et al (2007) Atomic structures of amyloid cross-beta spines reveal varied steric zippers. Nature 447: 453–457. 10.1038/nature05695 17468747

[18] Amenitsch H, Benetti F, Ramos A, Legname G, Requena JR (2013) SAXS structural studies of PrP^Sc^ reveals ~11 diameter of basic double intertwined fibrils. Prion 7: 496–500. 10.4161/pri.27190 24247356 PMC4201618

[19] Skora L, Fonseca-Ornelas L, Hofele RV, Riedel D, Giller K, et al (2013) Burial of the polymorphic residue 129 in amyloid fibrils of prion stop mutants. J Biol Chem 288: 2994–3002. 10.1074/jbc.M112.423715 23209282 PMC3561524

[20] Müller H, Brener O, Andreoletti O, Piechatzek T, Willbold D, et al (2014) Progress towards structural understanding of infectious sheep PrP-amyloid. Prion 8: 344–358. 10.4161/19336896.2014.983754 25482596 PMC4601355

[21] Meier BH, Böckmann A (2015) The structure of fibrils from 'misfolded' proteins. Curr Opin Struct Biol 30: 43–49. 10.1016/j.sbi.2014.12.001 25544255

[22] Wasmer C, Lange A, Van Melckebeke H, Siemer AB, Riek R, et al (2008) Amyloid fibrils of the HET-s(218–289) prion form a beta solenoid with a triangular hydrophobic core. Science 319: 1523–1526. 10.1126/science.1151839 18339938

[23] Gorkovskiy A, Thurber KR, Tycko R, Wickner RB (2014) Locating folds of the in-register parallel β-sheet of the Sup35p prion domain infectious amyloid. Proc Natl Acad Sci U S A 111: E4615–E4622. 10.1073/pnas.1417974111 25313080 PMC4217437

[24] Tuttle MD, Comellas G, Nieuwkoop AJ, Covell DJ, Berthold DA, et al (2016) Solid-state NMR structure of a pathogenic fibril of full-length human α-synuclein. Nat Struct Mol Biol 23: 409–415. 10.1038/nsmb.3194 27018801 PMC5034296

[25] Wälti MA, Ravotti F, Arai H, Glabe CG, Wall JS, et al (2016) Atomic-resolution structure of a disease-relevant Aβ(1–42) amyloid fibril. Proc Natl Acad Sci U S A 113: E4976–E4984. 10.1073/pnas.1600749113 27469165 PMC5003276

[26] Helmus JJ, Surewicz K, Nadaud PS, Surewicz WK, Jaroniec CP (2008) Molecular conformation and dynamics of the Y145Stop variant of human prion protein in amyloid fibrils. Proc Natl Acad Sci U S A 105: 6284–6289. 10.1073/pnas.0711716105 18436646 PMC2359773

[27] Zweckstetter M (2013) Conserved amyloid core structure of stop mutants of the human prion protein. Prion 7: 193–197. 10.4161/pri.23956 23406905 PMC3783102

[28] Smirnovas V, Baron GS, Offerdahl DK, Raymond GJ, Caughey B, et al (2011) Structural organization of brain-derived mammalian prions examined by hydrogen-deuterium exchange. Nat Struct Mol Biol 18: 504–506. 10.1038/nsmb.2035 21441913 PMC3379881

[29] Noble GP, Wang DW, Walsh DJ, Barone JR, Miller MB, et al (2015) A structural and functional comparison between infectious and non-infectious autocatalytic recombinant PrP conformers. PLoS Pathog 11: e1005017 10.1371/journal.ppat.1005017 26125623 PMC4488359

[30] Liu F, Heck AJ (2015) Interrogating the architecture of protein assemblies and protein interaction networks by cross-linking mass spectrometry. Curr Opin Struct Biol 35: 100–108. 10.1016/j.sbi.2015.10.006 26615471

[31] Gong B, Ramos A, Vázquez-Fernández E, Silva CJ, Alonso J, et al (2011) Probing structural differences between PrP^C^ and PrP^Sc^ by surface nitration and acetylation: evidence of conformational change in the C-terminus. Biochem 50: 4963–4972. 21526750 10.1021/bi102073j

[32] Onisko B, Guitián Fernández E, Louro Freire M, Schwarz A, Baier M, et al (2005) Probing PrP^Sc^ structure using chemical cross-linking and mass spectrometry: evidence of the proximity of Gly90 amino termini in the PrP 27–30 aggregate. Biochem 44: 10100–10109. 16042387 10.1021/bi0501582

